# Development and Psychometric Properties of a Scale to Measure the Meaning of Life (MLS)

**DOI:** 10.3390/ejihpe15090174

**Published:** 2025-08-29

**Authors:** Esvin Aldair Guevara-Tantalean, Anthony Brayham Tantaleán-Arteaga, Bruno Francesco Arévalo-García, Denis Frank Cunza-Aranzábal

**Affiliations:** Facultad de Ciencias de la Salud, Universidad Peruana Unión, Tarapoto 150118, Peru; esvinguevara@upeu.edu.pe (E.A.G.-T.); anthonytantalean@upeu.edu.pe (A.B.T.-A.); brunoarevalo@upeu.edu.pe (B.F.A.-G.)

**Keywords:** meaning of life, university students, psychometric, validity, reliability

## Abstract

The concept of meaning of life is of considerable significance to the Peruvian population, functioning as a protective factor that mitigates the occurrence of self-destructive behaviors. It constitutes a vital element of mental health, fostering personal development, adaptability to change, psychological well-being, life satisfaction, self-esteem, and optimism. The aim of this research was to develop and validate the Meaning of Life Scale (MLS) designed for the Peruvian population. This study involved 646 individuals aged between 18 and 69 years. One dimension was used, called the Presence of Life Meaning, and both exploratory factor analysis (EFA) and confirmatory factor analysis (CFA) were carried out, along with a reliability analysis. The results supported a unifactorial model with adequate indices (χ^2^(2) = 2.391, *p* < 0.001, CFI = 0.998, TLI = 0.995, RMSEA = 0.025, SRMR = 0.016) and high internal consistency (α = 0.878, ω = 0.878). The findings of this study offer preliminary evidence of the validity and reliability of the MLS.

## 1. Introduction

According to data from the World Health Organization, the global suicide mortality rate (per 100,000 inhabitants) is 9.2, whereas in Peru, it is 2.8 (WHO, 2024). However, this rate could be reduced even further if it is considered that meaning in life is a fundamental protective factor for the prevention of suicidal behavior (Lew et al., 2020). Additionally, according to the latest World Happiness Report, Peru ranks 58th [95% CI: 54–78] in reported happiness levels (WHR, 2024), a ranking that could be improved by understanding that meaning in life promotes happiness (King & Hicks, 2021).

Peru is often described as an “infinite country” due to its extensive geographical, cultural, and ethnic diversity, which contributes to its status as a nation of contrasts (Goldemberg & Zapata, 2009). In a nation where Andean and Amazonian communities coexist and interact, alongside the significant influence of Hispanic and, more broadly, Western cultures, emphasizing these distinctive attributes of Peru may promote the principles of solidarity, cooperation, and harmonious coexistence. Such an approach is crucial for addressing the country’s needs and engaging effectively within the context of globalization (Abanto-Chávez et al., 2023). Acknowledging the Peruvian diversity is considered essential for fostering a sense of purpose among individuals, thereby facilitating the development of their identity and sense of belonging. For instance, to contribute to this knowledge, the purpose of this study is to provide a new tool to measure the meaning of life in the Peruvian population.

The concept of meaning in life is defined as the perceived comprehension and significance that individuals attribute to the fundamental nature of their existence (Steger et al., 2006). This encompasses two dimensions of the existence of meaning in life: first, the presence of meaning, conceptualized as the individual’s sense of understanding themselves, their comprehension of the world, and their identification of their purpose within it; and second, the pursuit of meaning, which can be examined from two perspectives: as the absence or deficiency of meaning in life, and as the essential psychological motivation and necessity for human beings to comprehend their existence (Steger et al., 2008). According to Schnell (2009, 2021), the meaning of life can be conceptualized as the direction or purpose an individual pursues, along with the subsequent overall, subjective, and dynamic evaluation of their life as more or less meaningful. It comprises two dimensions: a positive experience of meaning, termed meaningfulness, and a negative experience, referred to as a crisis of meaning. However, as stated by King and Hicks (2021), there is an academic consensus that identifies three primary components of life’s meaning: comprehension or coherence, purpose, and mattering or significance. Comprehension or coherence pertains to an individual’s understanding of how elements of the past, present, and envisioned future are integrated into a coherent whole that imparts meaning to their life. Purpose refers to the perception that one’s actions are directed by valued goals. Mattering or significance involves an individual’s sense of importance based on the belief that their existence will have a lasting impact on the world, transcending time and space. However, Costin and Vignoles (2020) contend that mattering predicts both coherence and purpose, suggesting it is a distinct construct.

Baessler et al. (2003) assert that in Peru, the notion of meaning in life emphasizes growth as a crucial component. Ebersole (1998) defines this growth as the pursuit of personal goals and interests alongside maturity, self-improvement, personal worth, and independence. This conceptualization aligns with the widely held perspective that goals are a central element of life’s meaning (Schnell, 2009) as well as with the developmental and achievement dimensions associated with the source of meaning termed self-actualization (Damásio et al., 2013; Schnell, 2009).

**Importance of the Meaning of Life**

Meaning of life emerges as a fundamental protective factor that is intricately linked to resilience, mental health, and the ability to cope with adversity (Tsai et al., 2020). Beyond its significance for overall well-being, meaning of life is crucial in helping individuals confront challenging situations, particularly in those facing painful circumstances, such as the diagnosis of serious illnesses. It plays a vital role in alleviating emotional suffering, bolstering hope, and fostering a resilient attitude, which can positively influence both physical and mental recovery (Yousefi Afrashteh et al., 2023; Zhang et al., 2022).

The meaning of life serves as a crucial protective factor against suicidal behaviors by bolstering individuals’ resilience and enhancing their ability to cope with challenges (Lew et al., 2020). Similarly, its presence helps decrease the occurrence of self-destructive behaviors such as Internet addiction (González-Angulo et al., 2021). Consequently, the meaning of life is regarded as a vital component of mental health, as it correlates with reduced depression levels and heightened emotional well-being, fostering both personal growth and adaptability to change (Garrison & Lee, 2017; Maryam Hedayati & Mahmoud Khazaei, 2014). Moreover, its significance extends to the psychological well-being of older adults, where it is linked to increased life satisfaction, self-esteem, and optimism (Hallford et al., 2018).

A well-defined sense of purpose empowers individuals to tackle challenges more effectively and enhances their overall life satisfaction (Naghiyaee et al., 2020). A study conducted in Spain found that a clear meaning of life serves as a protective factor against suicidal ideation in patients with eating disorders (ED). Additionally, this sense of purpose was linked to increased resilience, which enabled these individuals to better manage the emotional and psychological challenges associated with their disorder (Balgiu, 2020).

Having a clear sense of purpose in life not only leads to having purposeful goals but also helps people feel more engaged with their daily actions. This, in turn, guides individuals to experience feelings of happiness and positive emotions, reinforcing the sense of purpose in life as a modulator when facing intense and complex experiences (King & Hicks, 2021). In fact, a study conducted among American physicians found that those who placed greater importance on the sense of purpose in life reported lower levels of burnout and fatigue, which was also associated with a better quality of life, serving as a protective factor against occupational exhaustion (Hooker et al., 2020). Additionally, people with routine jobs can find in leisure activities a way to reduce stress, thus generating a deeper and more stable sense of purpose in life (Hooker et al., 2020; Iso-Ahola & Baumeister, 2023).

**Measuring the meaning of life**

Several instruments have been developed to assess the meaning of life. The Purpose in Life Test (PIL), originally created in the United States, consists of 20 items, and evaluates two dimensions: the presence of purpose, which measures whether a person feels that their life has a clear purpose, and the search for purpose, which assesses the extent to which a person is actively seeking a purpose in life (Crumbaugh & Maholick, 1964). This test was validated and adapted in Colombia, showing a three-factor structure: goal-setting, affective satisfaction, and sense of achievement, while maintaining the original 20 items of the PIL instrument (Martínez Ortiz et al., 2012). In Argentina, with university students, confirmatory factor analysis confirmed a unifactorial model, indicating that it measures a single construct: purpose in life (Weber et al., 2022). In Spain, specifically in the province of Valencia, with a sample of people diagnosed with severe mental illness (SMI), a unifactorial model was also confirmed, demonstrating that the test measures a single construct: purpose in life (Rubio-Belmonte et al., 2024).

The Personal Meaning Index (PMI) is an instrument developed in Canada and is derived from the integration of two subscales of the Life Attitude Profile: purpose and coherence. Despite this, the PMI exhibits a unifactorial structure comprising 16 items (Reker, 2005). This measurement tool has experienced limited utilization outside its original developmental environment.

The Sources of Meaning and Meaning in Life Questionnaire (SoMe) is a German-origin scale comprising 151 items distributed across six dimensions. It evaluates 26 sources of meaning, categorized into four dimensions: self-transcendence, self-actualization, order and well-being, and relatedness. Furthermore, it assesses the meaning of life through two dimensions: meaningfulness and crisis of meaning (Schnell, 2009). This scale has been validated in Brazil (Damásio et al., 2013) and adapted to develop the Meaningful Work Scale, which consists of six items that characterize work experiences as fulfilling, significant, directed, coherent with life goals, and contributing to a sense of belonging (Schnell et al., 2013).

The Meaning in Life Questionnaire (MLQ), initially developed in the United States (Steger et al., 2006), comprises 10 items and is structured into two dimensions: the Presence of Meaning in Life, which assesses the extent to which an individual perceives their life as meaningful, and the Search for Meaning in Life, which evaluates the degree of active pursuit of meaning. These questionnaires have been validated in various contexts. The MLQ has demonstrated a two-factor structure, consisting of the Presence of Meaning in Life (MLQ-P) and the Search for Meaning in Life (MLQ-S), in various studies: in South Africa with a sample of university students (Temane et al., 2014), India with Hindi-speaking participants (Singh et al., 2016), Australia with a cohort of adolescents (Rose et al., 2017), Italy with an adult population (Negri et al., 2020), and in Peru with a population of university students (Travezaño et al., 2022). However, this latest version of the MLQ was validated in Peru only in a university population aged between 18 and 35 years. Additionally, it was based on an adaptation of the Argentine version rather than the original English version, recognizing cultural variability among Latin American countries and between these countries and the United States. Along these lines, a qualitative study that assessed the meaning of life using the Ebersole (1998) categorization of life meaning concepts reported that growth is an important component of the meaning of life in three out of four samples analyzed in Peru. This indicates that there is a particular conceptualization of the meaning of life in this country that requires further exploration (Baessler et al., 2003).

While various instruments have been developed, adapted, or validated across different countries, this study posits that cultural factors may not be adequately considered when instruments are translated and adapted to cultural contexts distinct from the original context in which they were constructed (Fernández et al., 2010). This assertion is grounded in the proposition that within a psychological framework, the psychological content of a dimension or trait cannot be indiscriminately generalized to a population different from that for which the instrument was originally designed, and even less so to populations from other countries or cultures (Matesanz, 1997). The validity of an instrument may be compromised when discrepancies arise in the interpretation of items due to cultural differences in the meanings of words, idiomatic expressions, and grammatical structures. Such discrepancies can result from a lack of experiential and conceptual equivalence between cultures, thereby altering participants’ understanding and responses to the items, ultimately affecting the accuracy of the measurements (Cha et al., 2007).

In the Peruvian context, there is a notable lack of instruments that have been specifically developed and psychometrically validated to assess the presence of meaning in life from a culturally grounded perspective. To date, the only available tool is an adapted and validated version of an instrument originally developed in a different cultural context. This research gap underscores the necessity to develop and validate an instrument tailored to the Peruvian context, facilitating an accurate and contextualized assessment of the meaning of life within this population. Consequently, the aim of the present study is to construct and validate a scale to measure the meaning of life in the adult Peruvian population, thereby providing the scientific community and mental health professionals with a valid and reliable instrument. Furthermore, this research seeks to advance the field of psychology in Peru by establishing a robust foundation for future interventions and studies related to the meaning of life.

## 2. Materials and Methods

### 2.1. Design

This study is categorized as quantitative, employing a survey-based, cross-sectional research design (Creswell & Creswell, 2023), and is characterized as a psychometric study as it focuses on the development, validation and evaluation of a measurement instrument, to ensure the reliability and validity of its measurements for their intended purpose (Rust et al., 2021).

### 2.2. Participants

A non-probabilistic or convenience sample approach was followed (Creswell & Creswell, 2023), collecting data according to the voluntary participation. Although the use of a single sample that is randomly divided into two subsamples is recommended in the scientific literature, another method involves collecting data in two stages—one for the EFA and the other for the CFA—which are carried out sequentially (Fletcher, 2023; Lloret-Segura et al., 2014). Therefore, the participants’ data were collected in two stages (n_1_ and n_2_), totaling 646 Peruvians. A Microsoft form was created in which the research instruments were included; then, the link to this form was sent individually to the researchers’ contacts through social networks such as WhatsApp, Facebook, and Instagram, asking them to forward it to their contacts in order to increase the sample size.

In the first sample (n_1_), ages ranged from 18 to 69 years old (M = 25.5; SD = 9.10). In the second sample (n_2_), ages ranged from 18 to 67 years old (M = 25.0; SD = 8.80). The sample size for the exploratory factor analysis (EFA: n_1_) was considered adequate as long as it exceeded the recommended minimum (n > 200), resulting in a sample size of n_1_ = 344 participants. To calculate the size of the second sample (n_2_), intended for confirmatory factor analysis (CFA: n_2_), the number of observed and latent variables in the model obtained from the EFA was considered, along with the expected effect (λ = 0.10), the desired level of statistical significance (α = 0.05), and the statistical power (1 − β = 0.90), which led to a minimum sample size to detect effect of 199. However, a sample size greater than the recommended minimum was used (n_2_ = 302).

As can be seen in Table 1, both samples (n_1_ and n_2_) display a wide geographic variability, which is considered relevant due to the country’s cultural diversity.

### 2.3. Ethical Aspects

The study adhered to the ethical standards set forth by the Helsinki declaration (World Medical Association, 2013), ensuring the protection of privacy and confidentiality of personal information, as well as minimizing any potential impact on the physical, mental, and social well-being of participants. Additionally, the research received approval from the research ethics committee of the primary authors’ affiliated university (Reference 2024-CEB-FCS—UPeU-N°232).

### 2.4. Instruments

The Meaning in Life Scale (MLS) is an instrument designed to assess the presence of meaning in life among the Peruvian population aged 18 and older. The initial version of the instrument included 46 items with 5 Likert-type response options: 1 = totally disagree, 2 = disagree, 3 = neither agree nor disagree, 4 = agree, and 5 = totally agree. An example of an item is “I have my goals clear”.

Firstly, a new set of items was developed based on the concept of presence of meaning in life by Steger et al. (2006) interpreted from the Peruvian perspective of the authors. For the creation of items, a specification matrix was first designed, which included only one content area. Then, following Rust et al. (2021), 15 cognitive, 15 behavioral, and 15 emotional manifestations were assigned to this single content area. These manifestations refer to preliminary expressions of the construct that are theoretically grounded and are intended to be refined and converted into items in the final version of the instrument. Cognitive items were designed to reflect beliefs and perceptions of meaning; emotional items to capture affective experiences associated with meaning; and behavioral items to represent engagement in actions that express or reinforce meaning. This approach draws on theoretical contributions from Steger et al. (2006), who define presence of meaning as the extent to which individuals perceive their lives as purposeful, and coherent, as well as from a Peruvian perspective of meaning in life (Baessler et al., 2003). Secondly, it was established that the items would be written to be measured with a Likert rating scale with 5 response options: Totally disagree = 1, Disagree = 2, Neither agree nor disagree = 3, Agree = 4, Totally agree = 5. Thirdly, since this is a person-centered instrument (Rust et al., 2021), the items were written based on the content area and the assigned manifestations, resulting in a 46-item pilot instrument. To reduce the likelihood of acquiescence or extreme responses, the items were written in a clear, unambiguous, and specific manner. Likewise, with the purpose of reducing social desirability bias, the items were formulated in such a way as to request responses in a non-direct manner, whenever possible. In order to avoid reading fatigue, a maximum of 20 words per item was considered. Fourthly, instructions were assigned for completing the instrument. Subsequently, data analysis procedures were carried out.

Two instruments were selected to obtain evidence of convergent construct validity in relation to other variables, based on previous studies that demonstrate the association between meaning in life, life satisfaction, and overall well-being (Hallford et al., 2018; Travezaño et al., 2022), which are described below.

The Satisfaction with Life Scale (SWLS), initially developed by Diener et al. (1985), adapted into Spanish by Vázquez et al. (2013), and validated in Peru by Calderón-De La Cruz et al. (2018), was another instrument employed in this study. This scale is designed to assess life satisfaction among adults. It comprises five Likert-type items, each with five response options: 1 = strongly disagree, 2 = disagree, 3 = neither agree nor disagree, 4 = agree, and 5 = strongly agree. An example item from this scale is: “In most ways, my life is close to my ideal.” Concerning its psychometric properties, the unidimensional model demonstrated satisfactory fit indices (CFI = 0.998, RMSEA = 0.052, SRMR = 0.027).

A third instrument used was the General Wellbeing Index (WBI), originally developed by the World Health Organization Regional Office for Europe (1998), adapted into Spanish by Simancas-Pallares et al. (2016), and validated in Peru by Caycho-Rodríguez et al. (2020). Its purpose is to assess people’s subjective well-being. It consists of 5 items in a Likert format with 5 response options: 1 = totally disagree, 2 = disagree, 3 = neither agree nor disagree, 4 = agree, and 5 = totally agree. An example of an item from this scale is: “I have felt active and energetic.” Regarding its psychometric properties, the unidimensional model showed satisfactory fit indices (CFI = 0.994, RMSEA = 0.053, SRMR = 0.018).

### 2.5. Data Analysis

Following the development of the initial set of 46 items, a content validity analysis was performed by three judges, who are experts in psychometrics and psychology. These judges meticulously assessed each item based on four criteria: clarity, congruence, context, and construct coverage. Clarity involved determining if the item statement was easily understandable; congruence evaluated the item’s alignment with the construct being measured; context examined the commonality of the language used within the study’s population; and construct coverage verified whether the item effectively assessed the specific component or dimension of the construct, which was considered unidimensional in this study. The evaluations provided by the judges were analyzed using Aiken’s V coefficient (Aiken, 2003). Items whose lower confidence interval limit was greater than 0.5 were considered valid (Penfield & Giacobbi, 2004).

The data were collected in two stages, one for exploratory factor analysis (EFA) and the second for confirmatory factor analysis (CFA). First, a descriptive analysis of the items was carried out, obtaining the mean, standard deviation, maximum and minimum values, as well as measures of skewness and kurtosis. Values within the ±2 range for both measures (EFA and CFA) were considered normally distributed (Bandalos & Finney, 2019).

Next, the items of the EFA sample were analyzed using the minimum residual extraction method and oblimin rotation, while the number of factors was determined through parallel analysis. Additionally, the assumptions for sample adequacy were verified using the Kaiser-Meyer-Olkin index (KMO > 0.9) and Bartlett’s test of sphericity (*p* < 0.001), considering only factor loadings greater than 0.4 and a minimum of three items per factor (Lloret-Segura et al., 2014; Nunnally & Bernstein, 1994).

Using data from the second sample, a confirmatory factor analysis (CFA) was conducted. The analysis was performed considering that if the assumptions of univariate normality are not satisfied, the maximum likelihood (ML) estimator should not be employed for confirmatory factor analysis (CFA). Instead, ML-Mean-adjusted χ^2^ and S-B scaled standard errors (MLM) estimator is recommended (Finney & DiStefano, 2013; Satorra & Bentler, 1994). This method is applicable to Likert-type scales with five response options (Rhemtulla et al., 2012).

The fit of the CFA model was evaluated using the chi-square (χ^2^) test; however, given the sensitivity of this test to sample size, more precise fit indices were considered, including the Comparative Fit Index (CFI), the Tucker–Lewis Index (TLI), the Root Mean Square Error of Approximation (RMSEA), and the Standardized Root Mean Square Residual (SRMR). CFI and TLI values greater than 0.90 indicate an acceptable fit, while values exceeding 0.95 denote a good fit (Kline, 2023; Schumacker & Lomax, 2016). For RMSEA and SRMR, values below 0.05 indicate a good fit, and values below 0.08 are deemed acceptable (Bandalos & Finney, 2019; Kline, 2023). Additionally, convergent construct validity was estimated through the Average Variance Extracted (AVE), with values above 0.50 considered adequate (Hair et al., 2019). Modification indices (threshold = 10) were used to refine the model by retaining only one item from each pair with correlated residuals, prioritizing those with higher factor loadings (λ). Items with λ > 0.70 were kept for the final version of the instrument. Consequently, the final set of retained items likely reflects the most central dimension of the construct (Clark & Watson, 1995). The reliability of the scale was verified using internal consistency indices: Cronbach’s alpha, McDonald’s omega, and H index. α, ω, H > 0.7 were considered acceptable values.

The convergent validity or validity evidence in relation to other variables (American Educational Research Association et al., 2018; VandenBos & American Psychological Association, 2015), specifically life satisfaction and general well-being, was evaluated using a structural equation modeling (SEM) approach, employing the same methods and fit indices considered for the confirmatory factor analysis (CFA) with data from the second sample (n_2_ = 302).

## 3. Results

### 3.1. Content Validity Analysis

The initial set of 46 items was evaluated by a panel of three experts. The experts’ responses were subsequently analyzed using Aiken’s V coefficient, resulting in acceptable values of V_Minimum_ [CI: 95%] = 0.83 [0.61; 0.64] and V_Maximum_ [CI: 95%] = 1.00 [0.82; 1.00] for only 18 items. These items were then selected for further stages of psychometric analysis (Supplementary Material Tables S1 and S2)

### 3.2. Preliminary Analysis

Descriptive statistics were computed for the 18 items across both samples: 344 for the EFA and 302 for the CFA (Table 2). For each item, the mean, standard deviation, skewness (g_1_), and kurtosis (g_2_) were determined. The skewness and kurtosis indices fell within the ±2 range, which is deemed acceptable (Finney & DiStefano, 2013) though some items surpassed this threshold (items 6, 8 and 16), indicating that the assumption of univariate normality required for the CFA with ML was not totally satisfied; consequently, the MLM estimator was deemed appropriate.

### 3.3. Preliminary Evidence of Internal Structure Validity

The sample for the Exploratory Factor Analysis (EFA) (n = 344) demonstrated appropriateness for EFA (Supplementary Material Table S3), as indicated by the Kaiser–Meyer–Olkin index (KMO = 0.969) and a significant Bartlett’s test of sphericity (*p* < 0.001). The EFA was conducted using the minimum residual extraction method and oblimin rotation. Items were retained if they exhibited a factor loading of 0.4 or higher and did not display factorial complexity that could hinder interpretation. Items 10 to 18 were grouped into a first factor, items 1 to 6 formed a second factor, while items 7 and 9 formed a third factor. It was decided to eliminate items 7 and 9 because a minimum of three items per factor was assumed. Ultimately, 16 items were identified, and subsequent parallel analysis suggested the presence of a single factor, termed “Presence of life meaning”.

### 3.4. Validity of the Internal Structure and Reliability

Table 3 presents the factorial structure of the 16 items derived from the exploratory factor analysis (EFA). These items were subsequently subjected to confirmatory factor analysis (CFA) (Supplementary Material Table S4); however, the fit indices were not satisfactory (χ^2^(104) = 322.899, *p* < 0.001, CFI = 0.848, TLI = 0.824, RMSEA = 0.083, SRMR = 0.064). Consequently, an analysis of modification indices was conducted using a threshold value of 10. For each pair of items exhibiting correlated residuals, only the item with the higher standardized factor loading (λ) was retained. Furthermore, only items with λ values greater than 0.70 were included in the final model. This process resulted in a refined model consisting of four items loading onto a single latent factor, which demonstrated adequate fit indices (χ^2^(2) = 2.391, *p* < 0.001, CFI = 0.998, TLI = 0.995, RMSEA = 0.025, SRMR = 0.016). This final model also provided evidence of convergent validity (AVE > 0.5) and internal consistency, with a satisfactory level of reliability (α = 0.878, ω = 0.878).

### 3.5. Evidence of Convergent Validity or Validity in Relation to Other Constructs

**Figure 1:**
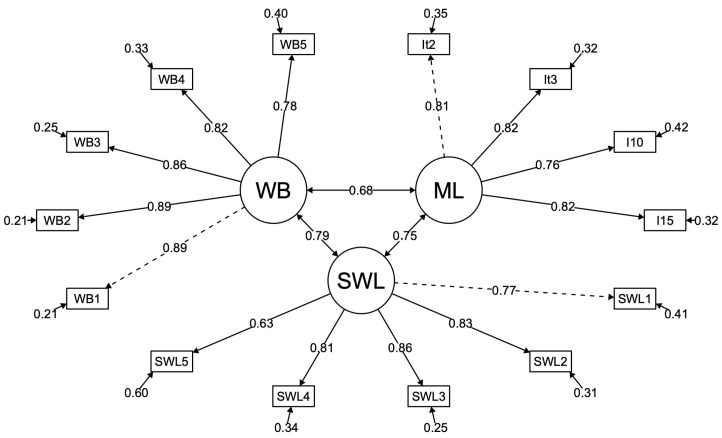
SEM model of validity evidence in relation to other constructs. ML = meaning of life; SWL = satisfaction with life; WB = general well-being.

Utilizing data from sample n_2_ for CFA, a structural equation modeling (SEM) approach was employed to examine the interrelationships among the construct of meaning in life and the constructs of life satisfaction and general well-being (Figure 1). The model demonstrated satisfactory fit indices (χ^2^(74) = 89.564, *p* = 0.105, CFI = 0.989, TLI = 0.986, RMSEA = 0.026, SRMR = 0.036). These results provide evidence suggesting that an enhanced sense of meaning in life is associated with increased levels of life satisfaction (ϕ = 0.75) and general well-being (ϕ = 0.68), providing evidence of convergent validity.

## 4. Discussion

The meaning of life is conceptualized as the interpretation individuals attribute to their existence, encompassing the value they place on their being and the significance they derive from their life experiences. This concept is intricately connected to the human pursuit of well-being, goal setting, self-understanding, and the enhancement of social relationships. In the context of Peru, it is crucial to develop a culturally appropriate scale to evaluate this construct, thereby enabling a more profound comprehension that resonates with the country’s cultural nuances. The purpose of the current study was to create and assess the psychometric properties of a scale intended to measure the meaning of life in Peruvian adults.

The evidence for the internal structure of the Meaning in Life Scale (MLS) is based on an exploratory factor analysis (EFA), which revealed a unifactorial structure. Subsequently, confirmatory factor analysis (CFA) supported this initial structure, obtaining adequate fit indices that provide evidence for the validity of the internal structure of the proposed single-factor model in this study, consistent with the original definition of the construct (Steger et al., 2006). Additionally, the factor loadings were greater than 0.7, indicating a robust factorial structure, as the items are influenced homogeneously and strongly by the latent variable. Moreover, adequate values for internal consistency reliability were obtained. This unifactorial structure was also demonstrated for other instruments measuring meaning in life, such as the PIL validated in Argentina and Spain (Rubio-Belmonte et al., 2024; Weber et al., 2022), although it differs from the three-factor structure found for that instrument in Colombia (Martínez Ortiz et al., 2012), or the two-factor structure of the MLQ (Steger et al., 2006; Travezaño et al., 2022).

Regarding validity in relation to other constructs, the SEM model indicated a positive relationship between meaning in life and life satisfaction, as well as with overall well-being, which means that the greater the sense of meaning in life, the higher the levels of life satisfaction and general well-being tend to be. These results are consistent with previous studies that considered convergent validity (Travezaño et al., 2022) or the link of meaning in life with life satisfaction (Hallford et al., 2018) and with overall well-being (Victoriana et al., 2023) or psychological well-being (Garrison & Lee, 2017).

This study presents preliminary evidence supporting the establishment of the Meaning in Life Scale for Peruvian adults (MLS) as a valid instrument within the Peruvian context (Supplementary Material Tables S5 and S6).

### 4.1. Theoretical Implications

The concept of the meaning of life has been explored from various perspectives, with a consensus in the scientific literature identifying three core components: comprehension or coherence, purpose, and mattering (King & Hicks, 2021). However, empirical evidence suggests that mattering is a distinct construct that predicts both comprehension or coherence and purpose (Costin & Vignoles, 2020). In the present study, the Meaning in Life Scale (MLS) includes items that focus on coherence and purpose. Item 4, “I am a happy person with my life,” reflects coherence as it pertains to an integrated perception of the experiential aspects that confer meaning to life, facilitating the understanding that each moment contributes to the entirety of life, as reflected in item 3, “I make the most of every moment of my life.” Conversely, items 1, “My life has a clear purpose,” and 2, “I have my goals clear,” assess purpose, emphasizing the focus on goals that provide a sense of meaning in life. This aligns with the Peruvian perception of the meaning of life, which emphasizes personal goals and interests, as well as maturity, self-improvement, personal worth, and independence (Baessler et al., 2003; Ebersole, 1998), and corresponds with the definitions of comprehension and significance by Steger et al. (2008) and purpose as defined by Schnell (2009, 2021). Therefore, this scale effectively encapsulates the items that measure the meaning of life from a Peruvian perspective, as a construct distinct from mattering. Consequently, based on these findings, the meaning of life is defined as the direction or purpose pursued by an individual, manifested through personal goals and interests, along with the maturity, self-improvement, personal worth, and independence it fosters, culminating in a comprehensive and coherent evaluation of one’s existence.

### 4.2. Practical Implications

Based on the findings of this study, the Meaning in Life Scale (MLS) emerges as a promising tool for assessing meaning in life within the Peruvian population. Its brevity facilitates its concurrent application with other scales to explore the relationship between meaning in life and various variables, including motivation, well-being, mood states, mental health, healthy habits, self-destructive behaviors, and burnout, among others. Furthermore, the MLS can serve as a screening instrument for the rapid assessment of meaning in life in clinical settings, thereby enabling the evaluation of a client’s development and progress in psychotherapeutic contexts. Its validation in the elderly population is particularly pertinent, given the demonstrated positive correlation between meaning in life and self-esteem, life satisfaction, and optimism in this demographic (Hallford et al., 2018). As meaning in life is conceptualized as a synthesis of coherence and purpose, it is proposed that the MLS could be employed as a measure of population health, in relation to variables that facilitate the analysis of the country’s economic health, thereby supporting the implementation of public policies in the domains of health and the economy. While the initial psychometric results appear promising, researchers emphasize the need for additional research to further validate the findings reported in this study.

### 4.3. Limitations and Future Research

The present study acknowledges certain limitations that warrant consideration. Firstly, the study was conducted via an online survey, which restricted its scope by excluding individuals without internet access. This limitation is reflected in the low participation of individuals with lower educational levels and a higher participation of those with tertiary education. Consequently, it is recommended that future studies validate the instrument in printed forms and include individuals with lower educational levels. This approach would enable researchers to determine whether the MLS is applicable to these populations or whether its application is confined to populations with higher educational attainment. Additionally, concerning age, the mean was between 25.0 and 25.5 years, with a standard deviation ranging from 8.80 to 9.10, indicating a greater concentration of data around the ages of 20 to 40, and less participation from older adults. Therefore, it is suggested that the instrument be validated and employed in geriatric populations, as older adults require the establishment of short-term, value-centered goals. Moreover, the development of a scale to measure mattering is also promising, as mattering is regarded as an important construct because of its predictive capacity for meaning in life. A mattering scale would have significant potential applications for individuals of all ages, particularly young people and older adults, for whom it could enhance their sense of meaning in life by focusing on their transcendence and the legacy they leave for future generations.

To strengthen the practical evidence and external validity of the MLS, it is essential for future research to investigate its applicability and effectiveness in real-world settings. This investigation should include educational programs for university students, clinical environments, and community-based initiatives that aim to enhance individuals’ sense of meaning in life.

## 5. Conclusions

The development of the Meaning of Life Scale (MLS) represents a promising contribution to assessing this construct within the Peruvian context. It facilitates an understanding of the meaning of life through lenses of purpose and coherence, thereby encapsulating a crucial construct in a concise set of items. This scale contributes to the study of personal identity development by determining whether individuals possess goals and purposes that align with their lives, thereby fostering a sense of coherence and happiness through self-assessment. The findings not only provide a tool specifically tailored to the Peruvian context but also lay the groundwork for future research investigating the relationship between the presence of meaning of life and various psychological and sociocultural variables. It is anticipated that this scale will assist in formulating strategies aimed at enhancing well-being in the Peruvian population.

## Figures and Tables

**Table 1:** Descriptive analysis of the samples under study.

Variables	Categories	n_1_ = 344 (EFA)		n_2_ = 302 (CFA)	
		n	%	n	%
Gender	Female	217	63.10%	195	64.60%
	Male	127	36.90%	107	35.40%
Marital status	Married	37	10.80%	22	7.30%
	Cohabitant	18	5.20%	15	5.00%
	Divorced	3	0.90%	4	1.30%
	Single	285	82.80%	257	85.10%
	Widowed	1	0.30%	4	1.30%
Department of current residence	Amazonas	10	2.90%	15	5.00%
	Ancash	-	-	2	0.70%
	Apurímac	2	0.60%	-	-
	Ayacucho	1	0.30%	2	0.70%
	Cajamarca	25	7.30%	30	9.90%
	Callao (Constitutional Province)	-	-	2	0.70%
	Cusco	1	0.30%	-	-
	Huancavelica	1	0.30%	-	-
	Huánuco	1	0.30%	1	0.30%
	Ica	3	0.90%	1	0.30%
	Junín	1	0.30%	1	0.30%
	La Libertad	13	3.80%	6	2.00%
	Lambayeque	19	5.50%	6	2.00%
	Lima	41	11.90%	14	4.60%
	Loreto	3	0.90%	10	3.30%
	Madre de Dios	1	0.30%	1	0.30%
	Moquegua	-	-	1	0.30%
	Pasco	1	0.30%	-	-
	Piura	1	0.30%	2	0.70%
	Puno	-	-	2	0.70%
	San Martín	218	63.40%	197	65.20%
	Ucayali	2	0.60%	4	1.30%
	Tumbes	-	-	5	1.70%
Level of education	None	2	0.60%	4	1.30%
	Primary	5	1.50%	2	0.70%
	High school	34	9.90%	98	32.50%
	Technician	51	14.80%	35	11.60%
	University	252	73.30%	163	54.00%
Employment Status	Active	145	42.20%	108	35.8%
	Not active	27	7.80%	24	7.90%
	Student	172	50.00%	170	56.30%

**Table 2:** Descriptive statistics of the items.

	Sample for EFA				Sample for CFA			
	M	SD	g_1_	g_2_	M	SD	g_1_	g_2_
Item 1	3.96	1.10	−1.32	1.31	3.92	0.98	−1.08	1.18
Item 2	3.92	1.05	−1.13	1.00	3.97	0.89	−0.92	1.09
Item 3	3.93	1.09	−1.19	0.96	4.07	0.88	−1.02	1.12
Item 4	3.80	1.11	−0.93	0.34	3.92	0.94	−0.76	0.31
Item 5	3.73	1.15	−0.85	0.05	3.73	1.01	−0.65	0.03
Item 6	4.16	1.03	−1.60	2.44	4.10	0.83	−1.03	1.62
Item 7	3.85	1.13	−1.01	0.36	3.98	0.95	−0.97	0.76
Item 8	4.15	1.06	−1.60	2.23	4.27	0.83	−1.42	2.81
Item 9	3.92	1.12	−1.14	0.69	4.03	0.97	−1.13	1.14
Item 10	3.88	1.11	−0.94	0.27	4.04	0.98	−1.08	0.84
Item 11	3.97	1.10	−1.28	1.18	4.03	0.93	−1.11	1.31
Item 12	3.88	1.07	−1.14	1.01	3.99	0.92	−0.99	0.96
Item 13	3.83	1.08	−0.98	0.54	3.94	0.94	−0.90	0.78
Item 14	4.01	1.12	−1.30	1.14	4.11	0.90	−1.15	1.52
Item 15	3.92	1.09	−1.10	0.78	4.00	0.95	−1.04	1.08
Item 16	4.10	1.07	−1.48	1.82	4.14	0.89	−1.32	2.09
Item 17	3.95	1.14	−1.21	0.81	4.10	0.93	−1.17	1.47
Item 18	4.01	1.06	−1.30	1.41	4.12	0.91	−1.22	1.66

Note. M = mean; SD = standard deviation; g_1_ = skewness; g_2_ = kurtosis.

**Table 3:** Exploratory and confirmatory factor analysis.

Initial N°	EFA		CFA	Final N°
	Factor	h^2^	Factor	
Item 1	0.847	0.717	_	_
Item 2	0.880	0.774	0.808	Item 1
Item 3	0.853	0.728	0.846	Item 2
Item 4	0.871	0.758	_	_
Item 5	0.841	0.708	_	_
Item 6	0.851	0.725	_	_
Item 8	0.814	0.663	_	_
Item 10	0.831	0.691	0.770	Item 3
Item 11	0.836	0.699	_	_
Item 12	0.864	0.747	_	_
Item 13	0.860	0.740	_	_
Item 14	0.914	0.836	_	_
Item 15	0.901	0.812	0.792	Item 4
Item 16	0.893	0.797	_	_
Item 17	0.881	0.775	_	_
Item 18	0.830	0.689	_	_
% of variance	74.100		_	_
α	0.979		0.878	α
ω	0.979		0.878	ω
_	_		0.972	H
_	_		0.644	AVE

Note. α = Cronbach’s alpha, ω = McDonald’s omega, λ = factor loadings, h^2^ = communality, AVE = average variance extracted.

## Data Availability

The data can be requested from the authors by correspondence.

## Supplementary Materials

The following supporting information can be downloaded at: https://www.mdpi.com/article/10.3390/ejihpe15090174/s1, Table S1: Item reduction process-English, Table S2: Item reduction process-Spanish, Table S3: Covariance matrix EFA, Table S4: Covariance matrix CFA, Table S5: Meaning of Life Scale-Spanish; Table S6: Meaning of Life Scale-English.
