# Supplementary material for: Development and Psychometric Properties of a Scale to Measure the Meaning of Life (MLS)
Source: Eur J Investig Health Psychol Educ. 2025 Aug 29;15(9):174. doi: 10.3390/ejihpe15090174 (PMC12468523; doi:10.3390/ejihpe15090174)
Supplement: Supplementary file 1 [file ejihpe-15-00174-s001.zip › Table S1 Item reduction process-English.pdf]

Item reduction process of the MLS

Items from the First (pilot) version	Items after content validation	Items after Factor Analysis (EFA and CFA)
1. I think that life has meaning.		
2. I understand the meaning of life.	1.*	
3. I am searching for the meaning of my life.		
4. My life has a clear purpose.	2.*	1.*
5. I have my goals clear.	3.*	2.*
6. I am satisfied with my life.	4.*	
7. I have discovered the meaning of life.	5.*	
8. I believe that people seek meaning in their lives.		
9. I believe that I should live my life with optimism.	6.*	
10. I am in search of happiness.	7.*	
11. I have clear values and principles that guide my life.	8.*	
12. I believe that there is a meaning to my life.		
13. My life has been easy up until this moment.		
14. Life is a box of surprises.		
15. My life can be summed up in happy moments and problems.		
16. My life has meaning.		
17. I am searching for a purpose in my life.	9.*	
18. I know how to express my gratitude.		
19. In the end, I know that life comes full circle.		
20. My life means little to me.		
21. I believe that life is an adventure.		
22. I live my life to the fullest.		
23. I make the most of every moment of my life.	10.*	3.*

24. I urge more people to live their lives the way they want.	11. I urge more people to live their lives in the best way possible.	
25. Sometimes my behavior puts my life at risk.		
26. The life I have displeases me.		
27. You have to take risks to know what it means to live.		
28. Having a private life is the best option.		
29. I don't dare to do what I like for fear of failing in life.		
30. I behave as if I were someone who has clear goals in life.	12.*	
31. Each action is another weight for each of us.		
32. I feel satisfied with what I have achieved in life.	13.*	
33. I feel gratitude towards life.	14.*	
34. I am a happy person.	15. I am a happy person with my life.	4.*
35. I enjoy the little things in life.	16.*	
36. I want to experience the pleasure of living in peace.	17. I experience the pleasure of living in peace.	
37. I feel like my life is getting shorter.		
38. Life is full of emotions.	18. My life is full of emotions.	
39. It makes me anxious to feel that my life is slipping away.		
40. I hate having birthdays without having accomplished something in life.		
41. I feel angry seeing that others do enjoy their lives.		
42. My life exhausts me.		
43. I've already lost the will to live.		
44. I'm scared to know that my future depends on me.		
45. I have problems that prevent me from living a full life.		
46. I need another person to give meaning to my life.		

Note. *The wording of the items was preserved without modifications, although the numbering changed.
