# Supplementary material for: Development and Psychometric Properties of a Scale to Measure the Meaning of Life (MLS)
Source: Eur J Investig Health Psychol Educ. 2025 Aug 29;15(9):174. doi: 10.3390/ejihpe15090174 (PMC12468523; doi:10.3390/ejihpe15090174)
Supplement: Supplementary file 1 [file ejihpe-15-00174-s001.zip › Table S2 Item reduction process-Spanish.pdf]

Proceso de reducción de ítems de la ESV

Ítems de la primera versión (piloto)	Ítems después de la validación de contenido	Ítems después del Análisis Factorial (AFE y AFC)
1. Pienso que la vida tiene sentido.		
2. Entiendo el significado de la vida.	1.*	
3. Busco el significado de mi vida.		
4. Mi vida tiene un sentido claro.	2.*	1.*
5. Tengo mis metas claras.	3.*	2.*
6. Estoy satisfecho con mi vida.	4.*	
7. He descubierto el significado de la vida.	5.*	
8. Creo que las personas buscan un significado a su vida.		
9. Considero que debo vivir mi vida con optimismo.	6.*	
10. Estoy en búsqueda de la felicidad.	7.*	
11. Tengo valores y principios claros que rigen mi vida.	8.*	
12. Pienso que existe un significado para mi vida.		
13. Mi vida ha sido fácil hasta este momento.		
14. La vida es una caja de sorpresas.		
15. Mi vida se resume en momentos felices y problemas.		
16. Mi vida tiene un significado.		
17. Busco un propósito para mi vida.	9.*	
18. Sé cómo expresar mi gratitud.		
19. Al final sé que la vida da vueltas.		
20. Mi vida es poco importante para mí.		
21. Considero que la vida es una aventura.		
22. Llevo mi vida al límite.		
23. Aprovecho al máximo cada instante de mi vida.	10.*	3.*
24. Insto a que más personas vivan su vida como ellos quieren.	11. Insto a que más personas vivan su vida de la mejor manera.	
25. A veces mi conducta hace que ponga en riesgo mi vida.		

26. La vida que tengo me disgusta.		
27. Hay que arriesgar para saber que es vivir.		
28. Tener una vida reservada es la mejor opción.		
29. No me atrevo a hacer lo que me gusta por temor a fracasar en la vida.		
30. Me comporto como si fuera una persona que tiene metas claras en su vida.	12.*	
31. Cada acción es un peso más para cada uno.		
32. Me siento satisfecho con lo que he logrado en la vida.	13.*	
33. Siento gratitud hacia la vida.	14.*	
34. Soy una persona feliz.	15. Soy una persona feliz con mi vida.	4.
35. Disfruto de las pequeñas cosas de la vida.	16.*	
36. Quiero experimentar el placer de vivir en paz.	17. Experimento el placer de vivir en paz.	
37. Siento que mi vida se acorta.		
38. La vida está llena de emociones.	18. Mi vida está llena de emociones	
39. Me desespera sentir que mi vida se va acabando.		
40. Odio cumplir años sin haber logrado algo en la vida.		
41. Tengo enojo por ver que otros sí disfrutan de su vida.		
42. Mi vida me cansa.		
43. Ya perdí el gusto por vivir.		
44. Me da miedo saber que mi futuro depende de mí.		
45. Tengo problemas que me impiden vivir una vida plena.		
46. Necesito de otra persona para dar un significado a mi vida.		

Nota. *La redacción de los ítems se conservó sin modificaciones, aunque la numeración cambió.
