# Supplementary material for: Development and Psychometric Properties of a Scale to Measure the Meaning of Life (MLS)
Source: Eur J Investig Health Psychol Educ. 2025 Aug 29;15(9):174. doi: 10.3390/ejihpe15090174 (PMC12468523; doi:10.3390/ejihpe15090174)
Supplement: Supplementary file 1 [file ejihpe-15-00174-s001.zip › Table S3 Covariance matrix EFA.pdf]

Observed covariances (lower triangle) and correlations (upper triangle)

	Item1	Item2	Item3	Item4	Item5	Item6	Item7	Item8	Item9	Item10	Item11	Item12	Item13	Item14	Item15	Item16	Item17	Item18
Item1	1.205	0.856	0.771	0.733	0.759	0.757	0.524	0.718	0.590	0.670	0.708	0.658	0.697	0.739	0.719	0.751	0.717	0.691
Item2	0.988	1.105	0.798	0.794	0.811	0.769	0.506	0.711	0.597	0.715	0.699	0.725	0.723	0.778	0.774	0.752	0.743	0.696
Item3	0.924	0.916	1.194	0.776	0.780	0.743	0.535	0.705	0.561	0.687	0.665	0.746	0.729	0.757	0.757	0.695	0.741	0.669
Item4	0.890	0.923	0.937	1.222	0.795	0.721	0.467	0.670	0.579	0.722	0.693	0.733	0.794	0.778	0.826	0.762	0.741	0.687
Item5	0.954	0.976	0.976	1.007	1.312	0.693	0.494	0.641	0.550	0.667	0.635	0.739	0.728	0.774	0.758	0.693	0.735	0.662
Item6	0.852	0.829	0.833	0.817	0.814	1.052	0.620	0.804	0.658	0.675	0.773	0.693	0.691	0.782	0.707	0.754	0.718	0.726
Item7	0.653	0.603	0.664	0.585	0.642	0.721	1.287	0.590	0.696	0.473	0.573	0.539	0.496	0.531	0.483	0.526	0.506	0.512
Item8	0.837	0.793	0.818	0.786	0.779	0.876	0.711	1.128	0.664	0.639	0.757	0.689	0.671	0.726	0.674	0.727	0.703	0.722
Item9	0.726	0.703	0.686	0.717	0.705	0.756	0.884	0.790	1.254	0.564	0.668	0.620	0.523	0.616	0.585	0.637	0.590	0.631
Item10	0.817	0.836	0.834	0.887	0.849	0.770	0.597	0.754	0.702	1.235	0.736	0.767	0.781	0.745	0.736	0.783	0.726	0.698
Item11	0.853	0.806	0.797	0.840	0.798	0.870	0.713	0.882	0.820	0.898	1.203	0.733	0.690	0.761	0.723	0.775	0.711	0.761
Item12	0.770	0.812	0.869	0.864	0.902	0.758	0.652	0.780	0.740	0.909	0.857	1.136	0.789	0.816	0.812	0.750	0.771	0.725
Item13	0.830	0.823	0.863	0.951	0.904	0.769	0.610	0.773	0.634	0.941	0.821	0.911	1.175	0.800	0.808	0.772	0.757	0.665
Item14	0.910	0.916	0.928	0.964	0.994	0.899	0.675	0.865	0.773	0.928	0.936	0.975	0.972	1.256	0.868	0.827	0.827	0.767
Item15	0.860	0.885	0.901	0.995	0.945	0.789	0.597	0.780	0.714	0.890	0.864	0.943	0.953	1.059	1.185	0.831	0.853	0.737
Item16	0.881	0.844	0.811	0.900	0.848	0.827	0.638	0.825	0.762	0.930	0.909	0.855	0.894	0.990	0.967	1.142	0.842	0.780
Item17	0.899	0.891	0.924	0.935	0.961	0.841	0.655	0.853	0.754	0.921	0.891	0.939	0.937	1.059	1.060	1.028	1.304	0.756
Item18	0.802	0.774	0.773	0.804	0.802	0.788	0.614	0.812	0.747	0.821	0.884	0.818	0.763	0.910	0.849	0.882	0.913	1.119
