# Supplementary material for: Development and Psychometric Properties of a Scale to Measure the Meaning of Life (MLS)
Source: Eur J Investig Health Psychol Educ. 2025 Aug 29;15(9):174. doi: 10.3390/ejihpe15090174 (PMC12468523; doi:10.3390/ejihpe15090174)
Supplement: Supplementary file 1 [file ejihpe-15-00174-s001.zip › Table S4 Covariance matrix CFA.pdf]

Observed covariances (lower triangle) and correlations (upper triangle)

	Item1	Item2	Item3	Item4	Item5	Item6	Item7	Item8	Item9	Item10	Item11	Item12	Item13	Item14	Item15	Item16	Item17	Item18
Item1	0.958	0.724	0.690	0.616	0.643	0.593	0.330	0.494	0.428	0.545	0.519	0.514	0.487	0.542	0.543	0.524	0.580	0.516
Item2	0.634	0.801	0.705	0.694	0.708	0.628	0.386	0.496	0.504	0.602	0.597	0.594	0.568	0.623	0.629	0.568	0.617	0.557
Item3	0.598	0.558	0.784	0.638	0.665	0.685	0.380	0.592	0.487	0.642	0.604	0.642	0.593	0.637	0.656	0.637	0.649	0.574
Item4	0.567	0.584	0.532	0.885	0.743	0.574	0.325	0.468	0.485	0.589	0.589	0.538	0.533	0.552	0.661	0.506	0.554	0.502
Item5	0.635	0.639	0.594	0.705	1.017	0.607	0.299	0.456	0.461	0.610	0.605	0.592	0.575	0.582	0.613	0.561	0.547	0.490
Item6	0.481	0.466	0.503	0.448	0.508	0.688	0.453	0.666	0.514	0.635	0.617	0.573	0.581	0.652	0.608	0.597	0.598	0.550
Item7	0.306	0.327	0.319	0.290	0.286	0.356	0.900	0.431	0.601	0.397	0.428	0.316	0.332	0.401	0.308	0.339	0.277	0.340
Item8	0.402	0.368	0.435	0.365	0.382	0.459	0.340	0.689	0.557	0.610	0.609	0.583	0.560	0.595	0.536	0.550	0.529	0.514
Item9	0.406	0.438	0.419	0.443	0.452	0.414	0.554	0.449	0.943	0.576	0.658	0.515	0.393	0.529	0.469	0.518	0.474	0.506
Item10	0.526	0.531	0.560	0.546	0.607	0.519	0.372	0.499	0.552	0.972	0.727	0.681	0.556	0.691	0.645	0.614	0.596	0.630
Item11	0.473	0.498	0.498	0.516	0.568	0.477	0.378	0.471	0.595	0.668	0.866	0.706	0.459	0.650	0.598	0.592	0.567	0.520
Item12	0.466	0.493	0.527	0.470	0.555	0.441	0.278	0.449	0.464	0.623	0.609	0.861	0.621	0.701	0.659	0.621	0.647	0.562
Item13	0.449	0.478	0.494	0.472	0.546	0.453	0.297	0.438	0.359	0.516	0.403	0.543	0.887	0.686	0.683	0.589	0.579	0.593
Item14	0.479	0.503	0.509	0.469	0.530	0.488	0.343	0.446	0.464	0.615	0.546	0.587	0.583	0.815	0.801	0.818	0.754	0.714
Item15	0.507	0.536	0.553	0.593	0.589	0.480	0.278	0.424	0.434	0.606	0.530	0.583	0.613	0.689	0.907	0.790	0.779	0.688
Item16	0.458	0.454	0.504	0.425	0.505	0.442	0.288	0.408	0.449	0.541	0.492	0.514	0.496	0.659	0.672	0.798	0.794	0.762
Item17	0.527	0.512	0.533	0.484	0.513	0.460	0.244	0.407	0.428	0.546	0.490	0.557	0.506	0.632	0.689	0.659	0.862	0.779
Item18	0.459	0.453	0.462	0.430	0.449	0.415	0.294	0.388	0.447	0.565	0.440	0.474	0.507	0.586	0.596	0.619	0.657	0.827
