# Supplementary material for: Development and Psychometric Properties of a Scale to Measure the Meaning of Life (MLS)
Source: Eur J Investig Health Psychol Educ. 2025 Aug 29;15(9):174. doi: 10.3390/ejihpe15090174 (PMC12468523; doi:10.3390/ejihpe15090174)
Supplement: Supplementary file 1 [file ejihpe-15-00174-s001.zip › Table S5 Meaning of Life Scale-Spanish.pdf]

Escala de Sentido de la vida (ESV)

A continuación, encontrarás una serie de ítems sobre el sentido de la vida, marca el casillero según tu criterio:

- Totalmente en desacuerdo = 1
- En desacuerdo = 2
- Ni de acuerdo, ni en desacuerdo = 3
- De acuerdo = 4
- Totalmente de acuerdo = 5

1	Mi vida tiene un sentido claro.	1	2	3	4	5
2	Tengo mis metas claras.	1	2	3	4	5
3	Aprovecho al máximo cada instante de mi vida.	1	2	3	4	5
4	Soy una persona feliz con mi vida.	1	2	3	4	5
