# Supplementary material for: Development and Psychometric Properties of a Scale to Measure the Meaning of Life (MLS)
Source: Eur J Investig Health Psychol Educ. 2025 Aug 29;15(9):174. doi: 10.3390/ejihpe15090174 (PMC12468523; doi:10.3390/ejihpe15090174)
Supplement: Supplementary file 1 [file ejihpe-15-00174-s001.zip › Table S6 Meaning of Life Scale-English.pdf]

Meaning of Life Scale (MLS)

Below, you will find a series of items about the meaning of life. Please check the box according to your opinion:

- Totally disagree = 1
- Disagree = 2
- Neither agree nor disagree = 3
- Agree = 4
- Totally agree = 5

1	My life has a clear purpose.	1	2	3	4	5
2	I have my goals clear.	1	2	3	4	5
3	I make the most of every moment of my life.	1	2	3	4	5
4	I am a happy person with my life.	1	2	3	4	5
